# Rhabdomyolysis in COVID-19: Report of Four Cases

**DOI:** 10.7759/cureus.10686

**Published:** 2020-09-27

**Authors:** Balraj Singh, Parminder Kaur, Ashesha Mechineni, Michael Maroules

**Affiliations:** 1 Hematology/Oncology, Saint Joseph's University Medical Center, Paterson, USA; 2 Cardiology, Saint Joseph's University Medical Center, Paterson, USA; 3 Internal Medicine, Saint Joseph's University Medical Center, Paterson, USA; 4 Hematology/Oncology, Saint Joseph's University Medical Center, Paterson, USA

**Keywords:** covid 19, sars-cov-2 (severe acute respiratory syndrome coronavirus -2), rhabdomyolysis, viral myositis, coronavirus, coronavirus disease, atypical presentation

## Abstract

Coronavirus disease 2019 (COVID-19) is a global public health emergency. COVID-19 is most well known for affecting the respiratory system, although it can also result in several extrapulmonary manifestations. Limited literature is available regarding rhabdomyolysis in COVID-19. We report four cases of rhabdomyolysis in COVID-19 patients. High index of suspicion is required for the appropriate clinical scenario to recognize this life-threatening situation so that complications can be avoided

## Introduction

Severe acute respiratory syndrome coronavirus 2 (SARS-CoV-2), which is responsible for the coronavirus disease 2019 (COVID-19), has infected millions of people and has caused more than hundreds of thousands of deaths globally. COVID-19 can present with a spectrum of clinical manifestations, including fever, myalgia, cough, dyspnea, and less frequently headache, diarrhea, nausea, and vomiting [1]. Although respiratory symptoms predominate, widespread organ-specific manifestations of COVID-19 are increasingly being reported. We report four cases of rhabdomyolysis in COVID-19.

## Case presentation

COVID-19 was diagnosed by nasopharyngeal swab real-time reverse transcription polymerase chain reaction (RT-PCR). Relevant clinical characteristics and laboratory values of the four cases are summarized in Table 1. All patients had elevated creatine kinase at presentation

**Table 1:** Clinical characteristics and laboratory values of the four cases Reference ranges are as follows: CK 30-223 unit/L, white cell count 4.5-11 K/mm^3^, hemoglobin 12-16 g/dL, platelets 140-440 K/mm^3^, troponin less than 0.03 ng/ml, sodium 135-145 mEq/ L, potassium 3.5-5 mEq/L, phosphorous 2.5-5 mg/dL, BUN 7-23 mg/dL,  creatinine 0.6-1.30 mg/dL, aspartate transaminase 13-39 U/L, alanine transaminase 7-52 U/L, ESR 0-32 mm/hr, CRP less than 10 mg/L, ferritin 12-300 ng/mL, LDH 140-271 U/L, d-dimer less than 0.5, urinalysis blood negative, urinalysis red blood cells 0-3/HPF. CK, creatine kinase; BUN, blood urea nitrogen; AST, aspartate transaminase; ALT, alanine transaminase; ESR, erythrocyte sedimentation rate; CRP, C-reactive protein; LDH, lactate dehydrogenase; ND, not done; HPF, high power field

Variable	Case 1	Case 2	Case 3	Case 4
Age	67	39	43	70
Sex	Male	Male	Male	Male
History of alcohol abuse/substance abuse/trauma/exertion	None	None	None	None
Influenza/parainfluenza/enterovirus/adenovirus	Negative	Negative	Not done	Not done
Home medications	None	Amlodipine, clonidine, hydrochlorothiazide, furosemide	Hydralazine	None
Intubated	Yes	Yes	No	Yes
CK at presentation (U/L)	589	4,330	8,636	5,008
Peak CK	19,773	4,330	9,793	5,008
Day of hospitalization corresponding to peak CK	Day 4	Day 1	Day 2	Day 1
White cell count (K/mm^3^)	8.7	8.2	8.2	11.8
Hemoglobin (g/dL)	13.4	14.9	11.8	15.7
Platelets (K/mm^3^)	161	136	115	147
Troponin (ng/mL)	0.02	0.4	0.4	0.3
Sodium (mEq/L)	139	134	134	144
Potassium (mEq/L)	4	4	5.1	3.5
Phosphorous (mg/dL)	2.8	ND	8.8	3.5
BUN (mg/dL)	18	57	74	32
Creatinine (mg/dL)	1.16	3.8	20	1.68
Acute renal replacement therapy	Yes	No	No	No
AST (U/L)	46	131	142	179
ALT (U/L)	30	65	43	53
ESR (mm/hr)	53	43	84	63
CRP (mg/L)	157.9	85.5	249	363.3
Ferritin (ng/mL)	574	1,170	5,217	1,780
LDH (U/L)	459	907	478	1475
D-dimer	0.62	1.92	1.06	3.11
Urinalysis blood	ND	Large blood	ND	Large blood
Urinalysis red blood cells (per HPF)	ND	4-5	ND	No red blood cells
Outcome	Died	Died	Died	Died

Case 1 

**Figure 1:**
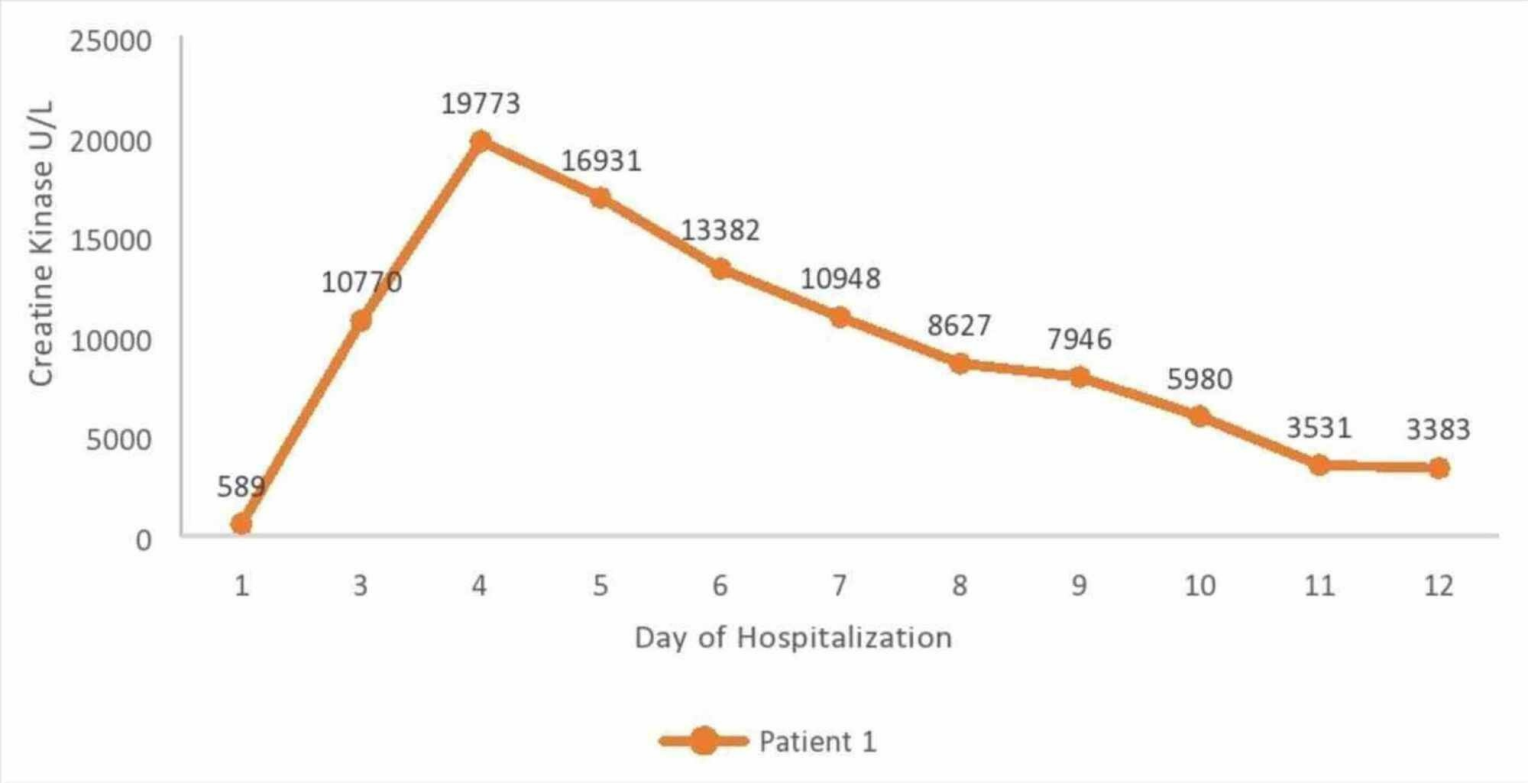
Creatine kinase levels corresponding to the day of hospitalization

A 67-year-old male patient with a past medical history of hypertension presented to the emergency department complaining of fever and shortness of breath. On initial examination, the patient was in severe respiratory distress, tachypneic, using accessory muscles, saturating at approximately 80% on room air, and was intubated. Figure 1 shows the creatine kinase levels corresponding to the day of hospitalization.

Chest X-ray performed at baseline showed bilateral hazy infiltrates. Chest CT showed extensive ground-glass opacity and consolidation in the lungs bilaterally. The patient was started on azithromycin, ceftriaxone, hydroxychloroquine, and fluids. On day 3, the patient developed acute renal failure and was started on hemodialysis. The patient had a complicated hospital course, requiring ventilatory support throughout the hospitalization and unfortunately passed away on day 21. 

Case 2 

A 39-year-old male patient with a history of hypertension presented to the emergency department with fever, muscle aches, shortness of breath, and altered mental status. On initial examination, the patient was in severe respiratory distress, tachypneic, using accessory muscles, hypoxic, and was intubated. Urinalysis was positive for large blood with four to five red blood cells (reference: 0-3) seen microscopically. Chest X-ray showed extensive confluent bilateral pulmonary infiltrates. The patient passed away due to cardiac arrest on day 1. 

Case 3 

**Figure 2:**
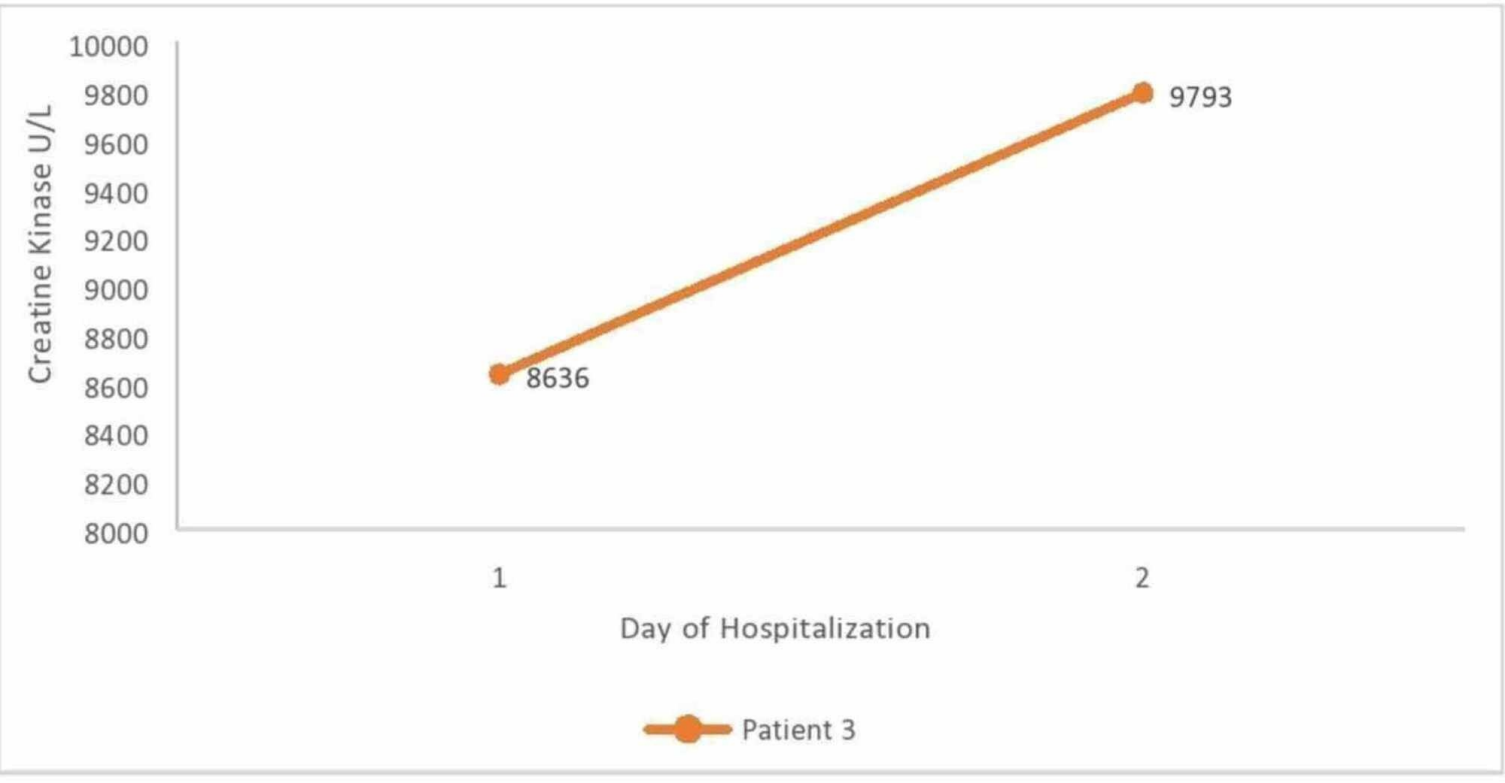
Creatine kinase levels corresponding to the day of hospitalization

A 43-year-old male patient with a past medical history of end-stage renal disease on hemodialysis comes to the emergency room with complaints of cough, shortness of breath muscle aches, and fever of two days of duration. Vital signs on presentation were heart rate of 107 beats per minute, blood pressure of 94/61 mm Hg, oxygen saturation 99% on room air, and temperature of 37.1 degree Celsius. On physical examination, diffuse bilateral rhonchi were present. Figure 2 shows the creatine kinase levels corresponding to the day of hospitalization.

Chest x ray showed multifocal ill-defined parenchymal opacities bilaterally, most notably over mid and lower lung zones with increased interstitial thickening. The patient was started ceftriaxone, hydroxychloroquine, and azithromycin. The patient passed away due to cardiac arrest on day 2. 

Case 4 

**Figure 3:**
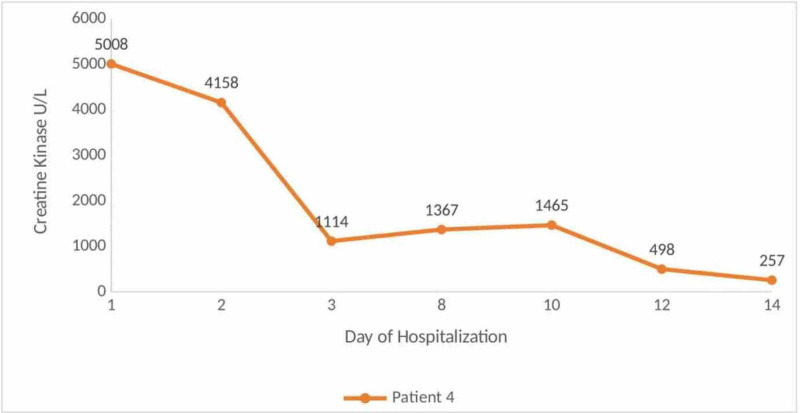
Creatine kinase levels corresponding to the day of hospitalization

A 70-year-old male patient with no significant past medical history presented to the emergency department with chief complaints of shortness of breath and cough of eight days of duration. Vital signs on presentation were heart rate of 101 beats per minute, blood pressure of 94/61 mm Hg, respiratory rate 20 breaths per minute, oxygen saturation 87% on room air, and temperature of 37.2 degree Celsius. The patient was intubated due to worsening respiratory status. Figure 3 shows the creatine kinase levels corresponding to the day of hospitalization.

Urinalysis was positive for large blood with no red blood cells seen microscopically. Chest x-ay showed bilateral hazy infiltrates throughout both lung fields. The patient was treated with fluids, intravenous methylprednisolone 40 mg q12 hours, and ceftriaxone. Hydroxychloroquine and azithromycin were not started due to prolonged corrected Q-T interval of 503 ms. The patient was extubated on day 8; however, he required reintubation on day 11. The patient had a complicated hospital course - septic shock, gastrointestinal bleed, and after discussion with the patient’s family, he was made comfort care and died on day 17.

## Discussion

Rhabdomyolysis is described as the rapid breakdown of skeletal muscles resulting in release of cell degradation products and intracellular elements within the bloodstream and extracellular space. Rhabdomyolysis can be caused by multiple etiologies, such as trauma, marked exertion in untrained individuals, hyperthermia, metabolic myopathies, drugs or toxins, infections, or electrolyte disorders [2]. The usual symptoms include muscle pain, weakness, and dark urine. The laboratory diagnosis is based on the measurement of creatine kinase in serum or plasma. A prompt diagnosis is a prerequisite for successful treatment and avoiding complications. Identification of the triggering event is of paramount importance. Early and aggressive fluid resuscitation is the cornerstone of management. Potential complications of rhabdomyolysis include fluid and electrolyte abnormalities, kidney failure, liver injury, cardiac arrhythmias, compartment syndrome, and disseminated intravascular coagulation [3].

Viruses that have been associated with rhabdomyolysis include influenza, parainfluenza, coxsackievirus, Epstein-Barr, herpes simplex, adenovirus, echovirus, human immunodeficiency virus, and cytomegalovirus [4,5]. Case reports of SARS-CoV-2 causing rhabdomyolysis have been described. Rhabdomyolysis has been described both as a presenting feature and a late complication of COVID-19. Chedid et al. described a case of a 51-year-old male patient with severe rhabdomyolysis leading to acute kidney injury (AKI) as the primary presenting feature of COVID-19 [6]. Jin and Tong reported a 60-year-old man with COVID-19, who was found to have rhabdomyolysis on day 9 of hospitalization when he developed pain and weakness of his lower extremities [7]. Possible mechanisms of action of viral-induced myositis include direct viral invasion or myotoxic cytokines or immune mediated [8,9]. Further studies are needed to understand the pathogenesis.

## Conclusions

We report four cases of rhabdomyolysis in COVID-19 patients. Our cases add to the limited literature regarding rhabdomyolysis in COVID-19. Clinicians should be aware of the life-threatening manifestation of COVID-19 so that timely diagnoses can be made, and complications avoided.
